# Liver Function Tests and FIB-4 Score as Predictors of Severity in COVID-19 Patients from the South-West of Romania

**DOI:** 10.3390/life12070934

**Published:** 2022-06-22

**Authors:** Adina M. Kamal, Florentina Dumitrescu, Adrian Mită, Denisa M. Săbiescu, Dragoș O. Alexandru, Codruța E. Gheorghe, Monalisa M. Filip, Adriana Ionescu-Ciocâlteu, Daniela T. Maria, Diana Kamal, Constantin K. Kamal

**Affiliations:** 1 Department of Internal Medicine, University of Medicine and Pharmacy of Craiova, 200349 Craiova, Romania; 2 Department of Infectious Disease, University of Medicine and Pharmacy of Craiova, 200349 Craiova, Romania; dumitrescu_florentina@yahoo.com (F.D.); denisa.sabiescu@yahoo.com (D.M.S.); 3 Department of Clinical Semiology, University of Medicine and Pharmacy of Craiova, 200349 Craiova, Romania; mitaadrian07@gmail.com; 4 Department of Informatics and Biostatistics, University of Medicine and Pharmacy of Craiova, 200349 Craiova, Romania; dragosado@yahoo.com; 5 Department of Family Medicine, University of Medicine and Pharmacy of Craiova, 200349 Craiova, Romania; constantinescu.codruta@yahoo.com (C.E.G.); kamalconstantin@gmail.com (C.K.K.); 6 Department of Gastroenterology, University of Medicine and Pharmacy of Craiova, 200349 Craiova, Romania; monalisafilip2007@gmail.com (M.M.F.); adriana_ciocalteu@yahoo.com (A.I.-C.); 7 Department of Nephrology, University of Medicine and Pharmacy of Craiova, 200349 Craiova, Romania; danagiurka@yahoo.com; 8 Department of Physical and Rehabilitation Medicine, Filantropia Municipal Hospital Craiova, 200516 Craiova, Romania; dianakamal84@gmail.com

**Keywords:** COVID-19, SARS-CoV-2, FIB-4 score, severity

## Abstract

Background: The coronavirus disease 2019 pandemic (COVID-19) is the most important global health crisis to date. In this study, we performed an analysis to find the association between liver damage, FIB-4 score and the severity of COVID-19 disease. Methods: We included a total of 580 patients that tested positive for SARS-CoV-2 infection and were hospitalized. No patient included had any known history of liver disease. Liver function tests were performed, and FIB-4 score was calculated in order to assess their involvement in the disease progression. Results: More than half of the patients had elevated liver function tests. Age, high body mass index, associated heart disease and diabetes were associated with poor outcome. Corticosteroids, antibiotics, and anticoagulants strongly correlated with liver injuries. Liver impairment and injury, as well as a FIB-4 score higher than 3.5, also correlated with higher degrees of disease severity. Conclusion: Liver injury and elevated FIB-4 score were associated with poor clinical outcome and disease severity, as well as being a valuable tool to predict COVID-19-related mortality.

## 1. Introduction

Respiratory and gastrointestinal diseases have been associated with a category of viruses from the coronavirus family [1]. There have been seven human coronaviruses discovered so far, including the virus that caused the most recent severe acute respiratory syndrome, coronavirus 2 (SARS-CoV-2) [2,3].

According to the most recent WHO figures, the virus has infected more than 268,189,593 people globally as of December 2021, killing 5,297,434 people. The World Health Organization (WHO) declared the disease a pandemic, resulting in thousands of fatalities and hospitalizations throughout the world [4,5,6,7]. Over half of COVID-19 patients had varied degrees of evidence indicating that on cholangiocytes the SARS-CoV-2 virus may adhere to the angiotensin 2 converting enzyme (ACE2), resulting in cholangiocyte malfunction and a systemic inflammatory response that leads to liver damage, according to new research [8]. Numerous studies have presented theories that certain variables may cause COVID-19-induced liver damage, which have been published since 10 March 2020 [9,10,11,12,13,14,15]. High amounts of GOT (ALT) and GPT (AST) were found in several trials, ranging from 14 percent to 53 percent [11]. A histological examination of liver biopsy specimens from a deceased COVID-19 patient demonstrated moderate microvesicular steatosis, as well as mild and portal lobular activity, implying that SARS-CoV-2 was responsible for the fibrosis and liver damage [12]. However, in Romania, there are few studies that have comprehensively analyzed liver enzyme values, the clinical features of liver failure, FIB-4 scores and the association of these changes with the risk of mortality and morbidity in patients with COVID-19.

## 2. Materials and Methods

### 2.1. Subject Characteristics

This was a cross-sectional retrospective study among patients recruited from the Infectious Disease and Pneumophtisiology “Victor Babes” Hospital Craiova, and the Clinical Municipal “Filantropia” Hospital Craiova, Romania. A total of 580 patients were enrolled in our study, starting January 2021 to December 2021. The criteria for enrolment were admission for COVID-19 and compliance to participation in study, and no history of liver disease. Before enrolling in the trial, all subjects were informed about the goal of the study and signed an informed consent form. Patients’ rights, outlined by the World Health Organization in the Patients’ Rights Act, 46/2003, were honored, considering human subjects participated in this study. We followed the Helsinki Declaration, which was established at the 18th World Medical Assembly in Helsinki in 1964 and updated at the 29th World Medical Assembly in Tokyo in 1975. This study was approved by the Ethics Committee of The University of Medicine and Pharmacy of Craiova (230/20 December 2021).

### 2.2. Patients

Out of the total included subjects, 305 were women (52.59%), and 275 (47.41%) were male. The youngest patient included was 17 years old, and the oldest was 91 years old, with a median of 56.46 years old across our cohort. The most encountered associated comorbidities were high blood pressure, type 2 diabetes (100 patients, 17.24%), chronic renal disease (38 patients, 6.55%), and cancer, with the most prevalent being hypertension with 255 (43.97) patients, and the least prevalent being cancer with 12 (2.07%) patients presenting neoplasia.

### 2.3. Confirmation of SARS-CoV-2 Infection

As per local guidelines of the Centre for Disease Control, the Public Health Organization, and the General Inspectorate for Emergency Situations in Romania, any patient presenting with upper respiratory infection exhibiting any of the following: fever, cough, shortness of breath without any other clear cause, or any patient with a radiological image suggestive of COVID-19, must be tested for the new coronavirus. The presence of SARS-CoV-2 infection was detected by real-time polymerase chain reaction (real time PCR) run with negative and positive control sets as suggested. 

### 2.4. Liver Damage Assessment 

Liver impairment was defined as the alteration above normal ranges for the liver function tests: ALT > 40 U/L, AST > 40 U/L, ALP (alkaline phosphatase) > 130 U/L, GGT (gamma-glutamyl transferase) > 48 U/L, total bilirubin > 1.1 mg/dL. Liver injury was defined as raised ALT, AST more than 3 × the upper limit unit of normal (ULN) ALP, GGT and total bilirubin more than 2 × the upper limit unit of normal (ULN). To check for liver fibrosis and risk stratification, we used a noninvasive test based on routine biochemical and clinical data (age, CBC, AST, ALT). FIB-4 is a reproducible score that has been found to be superior to other noninvasive fibrosis indicators, particularly in metabolic fatty liver disease [16,17].

The FIB-4 score uses the formula:

FIB-4 = Age (years) × AST (U/L)/[PLT (10^9^/L) × ALT^1/2^ (U/L)]

FIB-4 < 1.45 was considered within the normal range with a negative predictive value of advanced fibrosis of approximately 90% [16]; FIB-4 values greater than 3.25 were associated with a greater risk for developing advanced fibrosis, whereas FIB-4 values between 1.45 and 3.25 were considered intermediate risk.

### 2.5. Liver Test Abnormalities

We defined three categories according to liver test results. As previously mentioned, we defined the alteration of liver tests as ALT > 40 U/L, AST > 40 U/L, ALP (alkaline phosphatase) > 130 U/L, GGT (gamma-glutamyl transferase) > 48 U/L, and total bilirubin > 1.1 mg/dL. We classified the pattern as liver impairment, liver injury and no liver abnormalities. The liver impairment referred to the previously mentioned tests parameters and liver damage referred to ALT and/or AST levels three times over the normal value, as well as ALP, GGT, and/or TBIL levels exceeding two times the normal range.

### 2.6. Severity of Disease

Based on a physical exam, symptoms and radiologic findings, all patients were classified as severe or moderate cases according to the Romanian national guidelines for the management of the novel coronavirus. Non-severe types were defined as patients with minimal symptoms such as fever, expectoration, ageusia/anosmia, cough or other respiratory tract symptoms and a normal chest X-ray. A moderate alteration in chest X-ray was defined by the presence of multiple opacities or interstitial pneumonia, especially subpleural and in the marginal areas. The presence of any of the following traits were used to characterize severe pneumonia: (1) hypoxia: oxygen saturation (spontaneous) under 93%; (2) respiratory rate over 30 br/min, or the onset of respiratory failure or multiple organ failure, requiring admission to the Intensive Care Unit (ICU). 

### 2.7. Statistical Analysis

All the data were saved in Microsoft Excel files^®^ (Microsoft Corp., Redmond, WA, USA) and statistically evaluated to see if there was a link between a number of host-related parameters and hepatic impairment or injury. Excel^®^ was used to execute secondary data processing, such as computing essential statistical parameters, mean and standard deviation of their report, coefficient of variation, graphical depiction, and regression coefficient computation [18].

All data collected were processed using the Data Analysis ToolPak add-in, using chi tests, pivot tables, Pearson correlation coefficients; fundamental statistical parameters, mean and standard deviation, and coefficients of variation. The binary data were subjected to arcsine square root transformation to adjust for ANOVA analysis of count data instead of continuous variables.

## 3. Results

### 3.1. Baseline Characteristics and Clinical Features of Patients

Out of the total of 580 patients diagnosed with COVID-19 included in this study, 330 (56.89%) had elevated liver function tests (LFT). As defined previously, of these patients, 155 patients had liver injury and 175 had liver impairment. Of these 580 patients, 202 were asymptomatic or had mild disease, 183 had medium severity, and 195 developed severe or critical disease.

Data regarding baseline characteristics of subjects are presented in Table 1. 

The data in Table 1 shows that more than half of the patients admitted with COVID-19 had abnormal liver tests (56.8%), of which 30.17% had liver impairment as defined above, and 26.72% had liver injury. The majority of patients with abnormal liver tests were elderly and had high blood pressure and diabetes (*p* = 0.01), and had higher body mass indexes (BMI). In addition, most of the patients had cough (*p* = 0.03) and ageusia/anosmia (*p* = 0.001) as initial symptoms. 

### 3.2. Severity of Disease and Clinical Outcome

**Figure 1:**
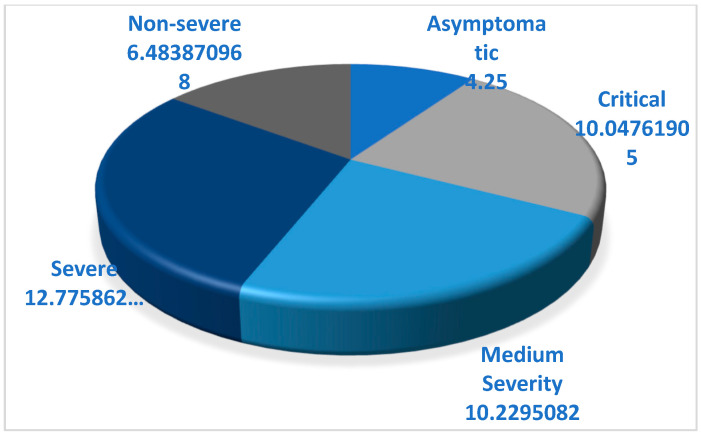
Average days of hospitalization according to the severity of disease.

Most of the patients admitted for COVID-19 with asymptomatic, mild, or non-severe disease had an average of 6.98 days of hospitalization, compared to 11.41 days for severe and critical disease (Figure 1). 

The most important radiology changes were observed for patients with liver injury, with 40.51% presenting with multiple opacities. Detailed data are presented in Table 2. 

**Figure 2:**
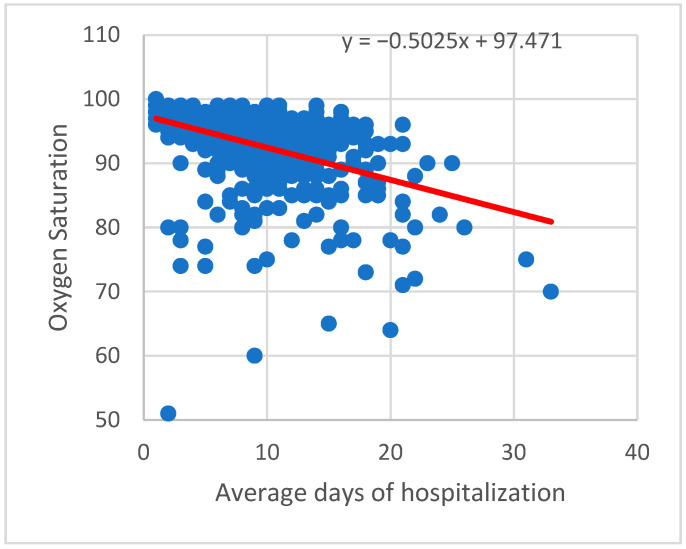
Average days of hospitalization correlated with oxygen saturation.

In addition, severity of disease and average days of hospitalization had strong correlations with oxygen saturation (SO_2_) at admittance; the lower the SO_2_, the higher risk of developing a more severe form (r Pearson= −0.42446, *p* < 0.001) (Figure 2).

Most patients with liver injury developed a severe and critical disease, and more than half of them died during the course of hospitalization (*p* > 0.001), thus explaining the high percentage of them needing ICU admittance. Patients treated with antivirals did not show an increased chance of developing severe disease compared to patients who were given antibiotics, anticoagulants, and corticosteroids (*p* < 0.001), with no other significant correlations regarding other specific treatments in our investigated lot. 

### 3.3. FIB-4 Association with Severity of Disease

Patients with a high risk of developing liver fibrosis were older (60–80+ years old, *p* < 0.01), had grade II or III obesity, and had associated renal disease and neoplasia (Table 3). 

**Figure 3:**
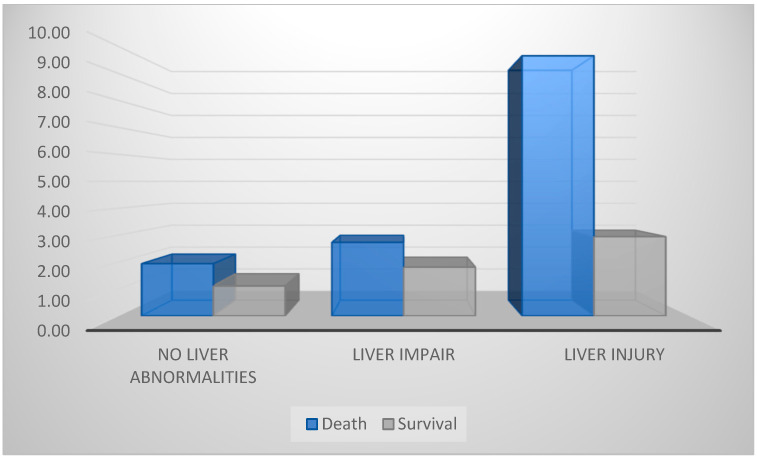
Survival according to FIB-4 score.

Difficulty breathing, cough and fever as the onset symptom were also associated with higher risk of developing liver fibrosis. In the survival group, we observed lower FIB-4 levels than in those in the death group, which recorded very high levels (Figure 3).

No significant differences were observed between women and men; additionally, no correlations could be made between D-dimers, troponin, procalcitonin, fibrinogen, C-reactive protein levels and FIB-4 score (R^2^ Pearson > 0.04). 

Drug use such as corticosteroids, favipiravir, molnupiravir and anticoagulants were found to correlate with a high risk of liver fibrosis. 

After adjusting for age, sex, BMI, associated pathology and use of drugs, FIB-4 score remained to have a high association with mortality.

## 4. Discussion

Our study is the first, and perhaps the most detailed, to describe the results of liver tests in COVID-19 patients in the south-west of Romania. Patients with abnormal liver test findings, especially those with liver injury, had significantly higher risks of developing severe disease than those who, at admission, had no changes in LFT. On admission, liver examination was performed for almost all subjects included in our study; therefore, anomalies in these tests can be utilized to predict disease severity. The leading cause of liver fibrosis risk or elevated liver function tests was the administration within the hospital of hepatotoxic drugs, especially favipiravir, molnupiravir, corticosteroids, antibiotics, and anticoagulants. Antibiotic use increased the odds of liver damage 2.13-fold, and anticoagulant use increased the odds 3.52-fold. As a result, patients who received these therapies should be continuously monitored, especially if they had abnormal liver test findings upon admission. Although our study included only patients with no known history of liver disease, the rate of LFT abnormalities was greater than anticipated, making us believe that COVID-19 could independently be the cause of these changes [19]. Many cell types and tissues, including the lungs, heart, blood vessels, kidneys, liver, and gastrointestinal system, contain ACE2, which was found to be the major receptor for entry into cells by SARS-CoV-2 in two recent studies [20,21]. It was recently discovered that SARS-CoV-2 can bond to cholangiocytes and cause liver injury [8], which could clarify how SARS-CoV-2 infection contributed to our patients’ LFT elevation. Additionally, the use of ACE inhibitors and ARBs may have an impact on liver testing. Overall frequency of elevated liver tests increased after admission, possibly as a result of more frequent exams and drugs administered. It is worth noting that just 7.07% of patients took lopinavir/ritonavir during their stay in the hospital, medicines that have been linked to liver injuries and abnormal LFT [22]. After admission, we noticed a significant increase in the incidence of liver injury. Approximately 51.72% of subjects in our study had abnormal liver tests while in the hospital. Because there were few extra variables influencing the abnormal liver test in these patients, we believe the disease is the most likely reason for this development. However, drug-induced liver impairment should be given more attention in hospitalized patients. Our research revealed that the use of corticosteroids, antibiotics and anticoagulants was associated with elevated risk of liver damage. These have previously been used to treat patients with SARS-23 and HIV-24 infections in China, and are widely used in Romanian patients with COVID-19. However, given the potentially high risk of liver injury, the efficacy and safety of these therapies warrant further investigation. Moreover, in China, drug-induced liver failure is most commonly reported with antibiotics and Chinese herbal medicine [23]. The present study suggested that the use of antibiotics, but not Chinese herbal medicines, was associated with liver injury in patients with SARS-CoV-2 infection. Patients with increased LFT levels, particularly those in the liver injury category, were considerably more prone to develop severe COVID-19, compared to those with normal tests. As previously stated in other studies, risk factors for developing severe SARS-CoV-2 infection have included variables like gender, age and patient history [15,24,25]. This is among the first publications in Romania’s southwestern region revealing abnormal liver tests linked to serious disease for this category of patients. SARS-CoV-2 is thought to be not only highly contagious, but also capable of causing multiorgan failure in humans [26,27], and our findings confirm this theory to some extent. There are some drawbacks to this study. For starters, because the patients in this study came from a single city, the findings cannot be applied to other areas with different epidemiological patterns. As additional cases appear around the world, further research in large groups of patients is needed. Furthermore, no information on additional causes of developing hepatotoxicity, such as herbal or other compounds used as a self-therapy, was available from our patients.

Nevertheless, since the outbreak of COVID-19 in December 2019, talks concerning the virus have spread swiftly online and have quickly become the center of worldwide interest. Because the internet is widely available, a large portion of the public will have higher cognizance on the matter. Because health insurance is widely available in Romania, the majority of patients (>56%) presented within 7 days of symptom-onset and seldom utilized over-the-counter drugs to self-treat. As a result, while data on herbal and other self-therapy were not available in our research, their impact on the outcomes may not be significant.

In this study, we also demonstrated that FIB-4 is strongly associated with clinical outcomes related to COVID-19. The direct virologic effect and inflammation could be the mediator, with the history of liver disease being a less probable factor. FIB-4 includes age and aspartate transaminase as operands, both of which are significantly linked with liver fibrosis [16,28,29]. It also mirrors the prevalent pattern of aspartate transaminase in COVID-19, previously detailed in other reports [30,31], as well as age, which was proven to be one of the most important indicators of poor outcome, as well as death, since the beginning of the pandemic [32].

Because of its qualities, the FIB-4 score is a more reliable predictor than any variable in its formula. Other non-invasive fibrosis ratings, such as aspartate aminotransferase to platelet ratio index, do not take age into account. Our results are similar to a recently published article from North America, in regard to COVID-19-related death prediction accuracy [33]. Our study shows that the spike in FIB-4 is likely due to various factors and related to the severity of COVID-19 disease. Skeletal muscle injury, increased pressure in the right cardiac system that led to liver stiffness, and portal system and hepatocellular alterations due to severe acute respiratory syndrome coronavirus 2 and inflammatory responses are all thought to have a role. First, we discovered that FIB-4 score is unrelated to troponin T levels, indicating that skeletal muscle injury might be a cause of AST and hence FIB-4 elevation. Second, greater right heart pressure has been linked to COVID-19 mortality in recent studies [34,35], leading us to believe that this might result in liver congestion, fibrosis, and injury. Third, direct virologic effects might explain why people who had a severe illness or died had consistently elevated FIB-4. We might need cohort follow-up studies to analyze the curve of FIB-4 score values at discharge and after disease resolution to determine whether the degree of liver fibrosis in the survival group maintains the same parameters or resolves after recovery. Two autopsy research studies have found that early signs of liver fibrosis are widespread in people who have died with COVID-19 [36,37]; one also found Kupfer cell activation, portal dilatation, and SARS-CoV-2 detection in the portal system in 68% of the specimens [38]. In conclusion, it is unclear if this is due to a pre-existing disease, direct virological effects, an inflammatory response, or a mix of these variables. To overcome this problem, new and more comprehensive pathology or autopsy studies are required. Because we omitted individuals with unknown hepatic disease, which can drastically determine the elevation of LFT or decrease of platelet count, there is no way to determine how the FIB-4 score might be a usable tool for prediction in patients with a history of hepatic disease. Our FIB-4 score research has a disadvantage in that it is uncertain as to how it might predict outcomes in people with a history of hepatic disease, who can have drastic elevations of AST, ALT, and low platelet count, because we omitted these patients with pre-existing liver disease from our analysis. In addition, as obesity was found in 150 patients in our study, it is unclear if these patients also had a non-alcoholic fatty liver disease, prior to admittance to the hospital for COVID-19. Testing for viral hepatitis, a thorough medical history with attention to living conditions and behavior, and hepatotoxic drug use should be provided prior to calculating the FIB-4 score, since all these variables could strongly impact its value and therefore can provide a false prediction value. Second, ultrasound elastography, Fibroscan^®^, or pathological examination [18] of the liver data are needed to establish whether an increased FIB-4 score is indeed associated with stiffness or fibrosis; therefore, more in-depth trials are needed to examine this [39]. Nonetheless, even with associated liver disease, and all the data we have available on SARS-CoV-2 regarding liver involvement, we can easily say that onset or progression of liver disease represents a risk factor for developing higher degrees of disease severity.

## 5. Conclusions

Finally, our findings suggest that elevated liver function tests and FIB-4 may play a prognostic role in COVID-19 patients. Our research found that this simple scoring system has high degrees of correlation to COVID-19-related mortality. More research is needed to confirm this conclusion in larger trials with elastography assessments, as well as to investigate precise tissue-level processes. Future research will be needed to determine if they truly account for liver fibrosis or if they are simply a COVID-19-induced alteration.

## Figures and Tables

**Table 1:** Baseline clinical features of patients with COVID-19.

	LIVER TESTS			
	No Liver Damage	Liver Impair	Liver Injury	*p*-Value
Number%	250 (43.10%)	175 (30.17%)	155 (26.72%)	
**Age**				
<30	13 (52%)	8 (32%)	4 (16%)	<0.001
30–39	31 (60.76%)	6 (11.76%)	14 (27.45%)	
40–49	59 (48.76%)	34 (28.10%)	28 (23.14%)	
50–59	53 (42.40%)	39 (31.20%)	33 (26.40%)	
60–69	50 (40.98%)	42 (34.43%)	30 (24.59%)	
70–79	32 (32.99%)	36 (37.11%)	29 (29.90%)	
80+	12 (30.77%)	10 (26.64%)	17 (43.59%)	
Sex				
Male	107 (38.91%)	93 (30.17%)	75 (26.72%)	0.1
Female	143 (76.89%)	82 (26.89%)	80 (26.23%)	
Associated pathology				
Hypertension	99 (38.82%)	74 (32.16%)	82 (29.02%)	0.1
Diabetes	38 (38%)	20 (20%)	42 (42%)	0.01
Renal disease	10 (26.32%)	14 (36.84%)	14 (36.84%)	0.08
Cancer	4 (33.33%)	2 (50%)	6 (16.67%)	0.3
Initial symptoms				0.3
Fever	136 (40%)	95 (32.06%)	109 (27.94%)	0.1
Fatigue	95 (42.22%)	64 (29.33%)	65 (28.44%)	0.75
Cough	174 (40.47%)	126 (30.23%)	130 (29.30%)	0.03
Difficulty breathing	40 (27.40%)	49 (39.04%)	57 (33.56%)	>0.001
Ageusia/Anosmia	71 (53.38%)	34 (21.05%)	28 (25.56%)	0.01
Chills	68 (43.59%)	41 (30.13%)	47 (26.28%)	
Body Mass Index (BMI)				
Normal	132 (53.66%)	73 (29.67%)	41 (16.67%)	<0.001
25–30	88 (47.83%)	49 (26.63%)	47 (25.54%)	
30–35	23 (27.06%)	38 (44.71%)	24 (28.24%)	
35–40	6 (13.95%)	13 (30.23%)	24 (66.81%)	
>40	1 (4.55%)	2 (9.09%)	19 (86.36%)	

**Table 2:** Severity and clinical outcome according to liver tests.

LIVER TESTS				
	No Liver Damage	Liver Impairment	Liver Injury	*p*-Value
Number %	250 (43.10%)	175 (30.17%)	155 (26.72%)	
Radiology changes				
0—no changes	111 (59.68%)	29 (24.73%)	46 (15.59%)	<0.001
1—interstitial	66 (40%)	53 (27.88%)	46 (32.12%)	
2—single opacity	36 (63.16%)	5 (28.07%)	16 (8.77%)	
3—multiple opacities	31 (19.62%)	64 (39.87%)	63 (40.51%)	
Severity of disease	111	29	46	
Asymptomatic	11 (68.75%)	5 (31.25%)	0 (0%)	<0.001
Non-severe	109 (58.60%)	48 (25.81%)	29 (15.59%)	
Medium severity	70 (38.25%)	58 (31.69%)	55 (30.05%)	
Severe	56 (32.18%)	57 (32.76%)	61 (35.06%)	
Critical	4 (19.05%)	10 (33.33%)	7 (47.62%)	
Clinical outcome				
Cured	89 (42.38%)	66 (31.43%)	55 (26.19%)	<0.001
Transfer to different ward	5 (41.67%)	5 (41.67%)	2 (16.67%)	
Not cured	151 (49.03%)	86 (27.92%)	71 (23.05%)	
Death	5 (10%)	18 (36%)	27 (54%)	
Drug use				
Antibiotics	181 (39.26%)	145 (31.45%)	135 (29.28%)	<0.001
Corticosteroids	138 (36.70%)	120 (31.91%)	118 (31.38%)	<0.001
Remdesivir	19 (35.19%)	16 (29.63%)	19 (35.19%)	0.29
Favipiravir	70 (42.45%)	50 (29.48%)	36 (28.06%)	0.48
Lopinavir/ Ritonavir	15 (43.60%)	15 (29.68%)	11 (26.72%)	0.75
Monoclonal antibodies	15 (43.28%)	14 (29.65%)	8 (27.07%)	0.5
Molnupiravir	66 (42.69%)	49 (29.23%)	34 (28.07%)	0.5
Anticoagulant	212 (40.30%)	168 (31.94%)	146 (27.76%)	<0.001
Intensive Care Unit (ICU) admittance	18 (26.87%)	16 (23.88%)	33 (49.25%)	<0.001

**Table 3:** FIB-4 score and baseline characteristics of cohort.

FIB-4 Score				
	No Liver Damage	Liver Impair	Liver Injury	*p*-Value
**Age**				
<30	0.52	0.57	0.77	<0.001
30–39	0.77	1.01	2.19	
40–49	0.94	1.28	2.93	
50–59	1.04	1.49	1.98	
60–69	1.39	2.20	4.57	
70–79	1.58	2.69	6.77	
80+	1.77	3.25	7.23	
Sex				
Male	1.20	2.09	3.73	0.01
Female	1.08	1.70	4.47	
Associated pathology				
Hypertension	1.13	1.91	4.11	0.04
Diabetes	1.39	2.52	4.92	0.02
Renal disease	1.56	2.41	8.46	<0.01
Cancer	1.15	2.85	9.56	<0.01
Initial symptoms				
Fever	1.18	1.69	4.43	0.01
Fatigue	1.18	2.07	3.46	0.4
Cough	1.14	1.93	4.34	0.04
Difficulty breathing	1.25	1.67	5.21	<0.001
Ageusia/ Anosmia	0.92	1.87	2.55	0.4
Chills	1.24	1.73	3.82	0.6
Radiology changes				
0—no changes	1.00	1.81	4.71	0.01
1—interstitial	1.19	1.83	4.42	0.01
2—single opacity	1.22	2.18	6.27	<0.01
3—multiple opacities	1.36	1.98	3.27	0.2
Severity of disease				
Asymptomatic	1.01	1.06	0	0.8
Non-severe	0.97	1.61	3.28	0.2
Medium severity	1.09	2.00	3.73	0.3
Severe	1.40	2.20	4.83	0.01
Critical	2.86	1.34	4.29	<0.01
Clinical outcome				
Cured	1.08	1.86	3.20	0.3
Transfer to different ward	1.92	1.89	2.47	0.4
Not cured	1.11	1.77	2.76	0.2
Death	1.94	2.73	9.65	<0.01
Drug use				
Antibiotics	1.18	2.02	3.84	0.2
Corticosteroids	1.18	1.91	4.05	0.01
Remdesivir	1.58	2.21	3.03	0.2
Favipiravir	1.21	1.81	5.01	0.01
Lopinavir/Ritonavir	1.25	1.47	3.26	0.3
Monoclonal antibodies	0.98	1.43	3.43	0.1
Molnupiravir	1.24	1.80	4.88	0.01
Anticoagulant	1.18	1.95	4.23	0.01
Intensive Care Unit (ICU) admittance	1.23	1.61	4.82	<0.01
Body Mass Index (BMI)				
Normal	1.04	1.91	2.66	<0.001
25–30	1.18	2.01	3.47	
30–35	1.49	1.82	2.76	
35–40	1.21	1.92	7.78	
>40	1.35	0.90	5.92	

## Data Availability

The data presented in this study are available on request from the corresponding author.
